# Undetected psychiatric morbidity among HIV/AIDS patients attending Comprehensive Care Clinic (CCC) in Nairobi Kenya: towards an integrated mental health care

**DOI:** 10.1186/s12991-018-0179-1

**Published:** 2018-03-02

**Authors:** Pauline W. Ng’ang’a, Muthoni Mathai, Anne Obondo, Teresia Mutavi, Manasi Kumar

**Affiliations:** 1 Department of Psychiatry, College of Health Sciences, University of Nairobi, P O Box 19676, Nairobi, 00202 Kenya; 2 Research Department of Clinical Health and Educational Psychology, University College London, London, WC1E 7HB UK

**Keywords:** HIV/AIDS, Psychiatric morbidity, Low-income status, Stigma

## Abstract

**Background:**

Psychiatric morbidity is commonly associated with HIV disease and may have adverse effects. This aspect may be overlooked at comprehensive HIV care centers in Low and Middle-Income Countries.

**Objectives:**

The aim of this study was to determine the prevalence of undetected psychiatric morbidity among HIV/AIDS adult patients attending Comprehensive Care Centre in a semi-urban clinic, in Nairobi, Kenya.

**Design:**

Descriptive cross-sectional study of adult HIV patients not receiving any psychiatric treatment was conducted.

**Participants/methods:**

The participants consisted of consecutive sample of adults (*n* = 245) attending HIV Comprehensive Care Clinic at Kangemi Health Centre, Nairobi. The Mini International Neuropsychiatric Interview (MINI) was administered to screen for undetected psychiatric morbidity. Socio-demographic characteristics were recorded in a questionnaire. Sample descriptive analysis was performed and prevalence of undetected psychiatric morbidity calculated. Chi-square test for independence was used to examine the associations between patient characteristics and undetected morbidity. Multivariable logistic regression analysis was performed to determine independent predictors of undetected psychiatric morbidity.

**Results:**

The mean age of our participants was 37.3 years (SD 9.2) Three-quarters (75.9%) of participants were females and median duration of HIV illness was 5 years. The prevalence of (previously undetected) psychiatric morbidity was 71.4% (95% CI 65.3–77). The leading psychiatric disorders were MDD (32.2%), PTSD (18.4%), Dysthymia (17.6%), and OCD (17.6%). Overall psychiatric morbidity was associated with low income (<USD 30), *p* = 0.035. MDD was associated with older age and female gender. There were no statistically significant associations between overall psychiatric morbidity and social determinants such as gender, marital status, level of education, religious affiliation, and occupation or employment status.

**Conclusion:**

The burden of psychiatric morbidity in Kenyan HIV patients remains high and is most significantly associated with lower socioeconomic status. There is need to provide holistic care including screening for mental well-being all through the treatment of HIV patients in low-income settings.

## Background

Several studies have consistently reported higher neuropsychiatric impairments in patients with HIV/AIDS [1]. Estimates from a systematic review of HIV-infected adults from Africa show that about one-half of HIV-infected adults have some form of psychiatric disorder [2]. People living with HIV/AIDS (PLWHA) are more susceptible to psychiatric morbidity compared to the general population [3] and major depressive disorder is the most common psychiatric diagnosis [4]. Other frequently reported psychiatric morbidities in HIV include suicidality, anxiety, post-traumatic stress disorder (PTSD), alcohol, and substance use disorders [5]. On one hand, psychiatric patients are at higher risk for acquiring HIV infection. On the other hand, patients infected with HIV are susceptible to developing psychiatric illness [6].

Despite the fact that sub-Saharan African (SSA) countries carry more than 90% of the burden of HIV/AIDS and studies report a high prevalence of mental health disorders related to HIV, common mental disorders often go undiagnosed and untreated in this population [7].

HIV-related deaths have declined significantly since the initiation of antiretroviral therapy (ART) worldwide in the year 1996 [8]. The reported success with scale-up of ART programs has brought to the fore the underlying burden of psychiatric morbidity in HIV/AIDS. Firstly, HIV/AIDS is now managed as a chronic illness using existing ARV drugs. While chronic illnesses are known risk factors for psychiatric morbidity, these psychiatric illnesses can also contribute to poor adherence and HIV treatment failure [9, 10]. Secondly, initial ARV guidelines recommended nucleoside reverse transcriptase inhibitors (NRTIs) and non- nucleoside reverse transcriptase inhibitors (NNRTIs) as first-line ARVs [11]. The association between NRTIs and NNRTIs with central nervous system manifestations including mania and psychosis led to significant increase in prevalence of undetected psychiatric morbidity [12]. These first-line ARVS were subsequently replaced by drugs with limited CNS effects.

In the Kenyan health care context, patients have limited access to mental health services and this is coupled with a shortage of mental health professionals, a fact that is documented globally about LMICs [13]. There have been specific efforts in Kenya to integrate mental healthcare into the management of PLWHA with national guidelines now recommending inclusion of psychosocial support, mental health screening, and management in the standard package of care for HIV. The National AIDS and STI control program (NASCOP) in Kenya recently launched new guidelines that recommend basic screening of depression before initiating ART and annual screening thereafter [14]. This study sought to determine the prevalence of undetected psychiatric morbidity among HIV/AIDS adult patients attending Comprehensive Care Centre (CCC) in a semi-urban clinic in Nairobi, Kenya.

## Methods

This was a descriptive, cross-sectional study conducted to determine the prevalence and types of previously undetected psychiatric morbidity in a low-income urban informal settlement population receiving comprehensive care for HIV/AIDS at a government-sponsored Health Centre in Nairobi from March to August, 2014. The clinic operates twice a week (Tuesdays and Thursdays) and serves approximately 80 eligible patients on each day. Patient selection was done through consecutive sampling of clients on arrival at the clinic. We recruited 245 participants based on a precision of 5% around an estimated prevalence of 80–90% for psychiatric disorders reported in HIV-positive adults by Musisi and Kinyanda [15]. Adult patients were eligible to participate if they were not on treatment for any psychiatric illness and provided consent. Patients were excluded if they were too ill.

The interviews were conducted at a Comprehensive Care Centre in Kangemi Health Centre by two research assistants along with the first author. This work contributed towards the first author’s dissertation for degree course in Masters of Medicine in Psychiatry at the University of Nairobi. The remaining authors acted as her mentors. The first author trained the assistants on administration of the Mini International Neuropsychiatric Interview (MINI plus), a diagnostic interview used for screening individuals for psychiatric diagnoses [16]. Individual interviews lasting between 30 and 45 min were carried out while the patients waited to be seen by the CCC medical officer. During the interview, each participant had the MINI plus administered and also completed a questionnaire on socio-demographic characteristics.

Data analysis was performed using IBM SPSS statistics for Windows, version 19 [17]. Descriptive analysis of sample characteristics was conducted by calculating means (SD) for continuous data and frequency summaries (counts and percentages) for categorical data. The main outcome was calculated as the proportion of HIV patients attending comprehensive care who screened positive for previously undetected psychiatric morbidity (using the MINI plus). Chi-square test for independence was used to examine associations between patient characteristics and undetected psychiatric morbidity. Similar analysis was conducted for each of the common psychiatric morbidities. Multivariable logistic regression analysis was then performed to determine independent predictors of undetected psychiatric morbidity. Variables that showed significant association with psychiatric morbidity in the Chi-square tests based on *p* value (< 0.05) were selected for inclusion in the logistic regression model as independent variables while the variable for prevalence of psychiatric morbidity was used as the dependent variable.

## Results

### Sample characteristics

**Fig. 1:**
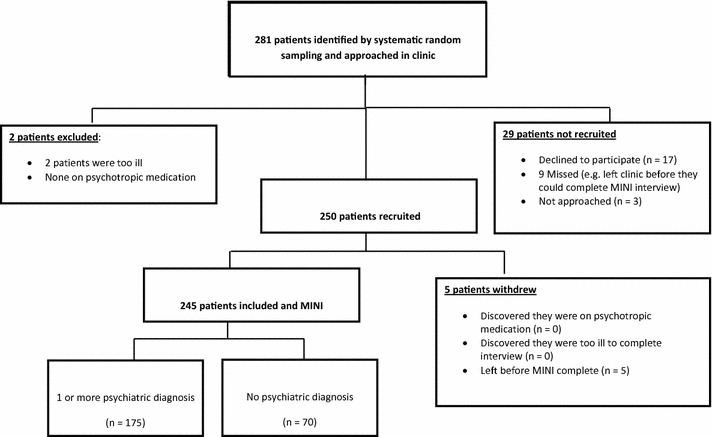
Participant recruitment flow chart

We recruited 281 HIV-positive adults in the study; however, only 245 were included in the final analysis. Thirty-six (36) patients were excluded during recruitment and data collection. This was due to illness, declining to participate after intake due to undisclosed reasons, others left before they could complete the MINI interview. See Fig. 1 which presents a flow chart of patient selection and recruitment.

The mean age (± SD) of participants was 37.3 years (± 9.2) with a range of 19–70 years. Of the 245 participants, 75.9% (*n* = 186) were females, 46.5% (*n* = 114) were married, and 55.1% (*n* = 135) had basic to no formal education (see Table 1). Most patients were unemployed 53.5% (*n* = 131), engaged as unskilled workers or casual workers 69.8% (*n* = 171), and 52.2% reported a monthly income < KES 3000 (approximately USD 30).

**Table 1:** Demographic characteristics and unidentified psychiatric morbidity in adult HIV/AIDS patients attending CCC in Nairobi suburbs

Socio-demographic characteristics	Total (*n* = 245)		Undetected psychiatric morbidity		*p* value
	*N*	%	Present (*n* = 175), *N* (*N* %)	Absent (*n* = 70), *N* (*N* %)	
Sex					
Male	59	24.1	38 (64.4)	21 (35.6)	0.17
Female	186	75.9	137 (73.7)	49 (26.3)	
Marital status					
Single	39	15.9	27 (69.2)	12 (30.8)	0.91
Married	114	46.5	81 (71.1)	33 (28.9)	
Separated (divorced/widowed)	92	37.6	67 (72.8)	25 (27.2)	
Level of education					
Primary or none	135	55.1	97 (71.9)	38 (28.1)	0.59
Secondary	89	36.3	65 (73.0)	24 (27.0)	
College/university	21	8.6	13 (61.9)	8 (38.1)	
Reported monthly income					
Less than KES 3000 (USD 30)	117	47.8	91 (77.8)	26 (22.2)	*0.03*
More than KES 3000 (USD 30)	128	52.2	84 (65.6)	44 (34.4)	
Religious affiliation					
Catholic	68	27.8	50 (73.5)	18 (26.5)	0.57
Protestant	162	66.1	116 (71.6)	46 (28.4)	
Other	15	6.1	9 (60.0)	6 (40.0)	
Currently employed					
Yes	114	46.5	77 (67.5)	37 (32.5)	0.21
No	131	53.5	98 (74.8)	33 (25.2)	
Current occupation					
Skilled	74	30.2	49 (66.2)	25 (33.8)	0.235
Unskilled	171	69.8	126 (73.7)	45 (26.3)	

### Burden of previously undetected psychiatric morbidity

We found that about 71.4% (175 out of 245 participants) of HIV-positive adults attending care had psychiatric morbidity detected through the MINI. The most common diagnoses were major depressive disorder (32.2%), post-traumatic stress disorder (18.4%), dysthymia (17.6%), obsessive compulsive disorder (17.6%), low-risk suicidality (16.3%), and mania (11.4%).

### Association between psychiatric morbidity and socio-demographic factors

A significant association between income status and presence of psychiatric morbidity, which was more prevalent among patients with low (< 30 USD) compared to high-income [*χ*^2^ = 4.4 (1), *p* = 0.03] participants was found. Though we did not find any significant associations between the presence of psychiatric morbidity and participant demographic characteristics such as sex, marital status, level of education, religious affiliation, and occupation or employment status (see Table 1).

### Duration of HIV illness, CD4, and partner attributes

The mean duration (± SD) of HIV illness was 5.7 years (± 4.1). Out of the 245 patients, 17.6% (*n* = 38) had CD4 counts < 200 cell/mm^3^, 18% (*n* = 44) reported that the partner had died, and 10.6% (*n* = 26) had a child with HIV-positive diagnosis. These factors did not show a significant association with prevalence of undetected psychiatric morbidity (see Table 2).

**Table 2:** Duration of HIV/AIDS diagnosis, CD4 count, partner status, and prevalence of unidentified psychiatric morbidity in adult HIV/AIDS patients attending CCC in Nairobi suburbs

Variables	*N*	%	Undetected psychiatric morbidity		*p* value
			Present (*n* = 175)	Absent (*n* = 70)	
CD 4 count (cells/mm^3^)					
> 200	178	82.4	122 (68.5)	56 (31.5)	0.532
< 200	38	17.6	28 (73.7)	10 (26.3)	
HIV duration (years)					
< 5	103	42	70 (68.0)	33 (32.0)	0.59
5–9	107	43.7	79 (73.8)	28 (26.2)	
10 years and above	35	14.3	26 (74.3)	9 (25.7)	
Child status					
Positive	26	10.6	21 (80.8)	5 (19.2)	0.10
Negative	171	69.8	122 (71.3)	49 (28.7)	
Unknown	26	10.6	14 (53.8)	12 (46.2)	
No children	22	9	18 (81.8)	4 (18.2)	
Partner died					
Yes	44	18	34 (77.3)	10 (22.7)	0.34
No	201	82	141 (70.1)	60 (29.9)	
Partner status HIV positive					
Yes	58	51.3	41 (70.7)	17 (29.3)	0.98
No	55	48.7	39 (70.9)	16 (29.1)	

### Previously undetected major depressive disorder (MDD) and its risk factors

Table 3 shows that undetected MDD occurred in older patients (mean age 39.4 versus 36.3 years, *p* = 0.013), and that MDD was more prevalent in females compared to males (36% versus 20.35, *p* = 0.025) and patients in households with lower monthly income (41 versus 21.3%, *p* = 0.005). There was no association between MDD and religion, marital and employment status, education, or occupation.

**Table 3:** Unidentified major depressive disorder in adult HIV/AIDS patients attending CCC in Nairobi suburbs

Variables	N	%	Undetected MDD		*p* value
			Presence	Absence	
Mean age (± SD), 37.3 (9.2)			39.4 (± 9.0)	36.3 (± 9.1)	0.013*
Gender					
Male	59	24.1	12 (20.3)	47 (79.7)	0.025*
Female	186	75.9	67 (36.0)	119 (64.0)	
Marital status					
Single	39	15.9	12 (30.8)	27 (69.2)	0.64
Married	114	46.5	34 (29.8)	80 (70.2)	
Separated (divorced or widowed)	92	37.6	33 (35.9)	59 (64.1)	
Level of education					
Primary or none	135	55.1	42 (31.1)	93 (68.9)	0.10
Secondary	89	36.3	34 (38.2)	55 (61.8)	
College/university	21	8.6	3 (14.3)	18 (85.7)	
Currently employed					
Yes	114	46.5	35 (30.7)	79 (69.3)	0.63
No	131	53.5	44 (33.6)	87 (66.4)	
Reported monthly income					
Less than KES 3000	117	47.8	48 (41.0)	69 (59.0)	0.005*
More than KES 3000	128	52.2	31 (24.2)	97 (75.8)	
Religious affiliation					
Catholic	68	27.8	19 (27.9)	49 (72.1)	0.325
Protestant	162	66.1	57 (35.2)	105 (64.8)	
Other	15	6.1	3 (20.0)	12 (80.0)	
Occupation					
Skilled	74	30.2	21 (28.4)	53 (71.6)	0.394
Unskilled	171	69.8	58 (33.9)	113 (66.1)	

*significant *p* value of less than 0.05

We did not conduct multivariable regression for overall prevalence of undetected psychiatric morbidity because none of the factors were associated with this outcome in the univariable analysis. Multivariate logistic regression with MDD as dependent variable and age, sex, and income as predictors was conducted. CD4, duration of HIV diagnosis, and formal education were included in the model as a priori confounders of the prevalence of undetected MDD. Table 4 shows that after adjusting for the effect of CD4, HIV illness duration, and education level, the odds of undetected MDD among females were three-fold higher compared to males (OR = 3.33; 95% CI 1.32–8.45) and these odds increased by 4% for unit increase in patients age (OR = 1.04; 1.00–1.08).

**Table 4:** Logistic regression of independent predictors of MDD in HIV/AIDS patients attending CCC in Nairobi suburbs

Variables	OR (95% CI)	*p* value
Age in years	1.04 (1.00–1.08)	0.031*
Sex		
Male	1.0 (ref)	
Female	3.33 (1.32–8.45)	0.011*
Monthly income		
Less than KES 3000	1.0 (ref)	
More than KES 3000	0.60 (0.31–1.14)	0.12
Level of education		
None or primary	1.0 (ref)	
Secondary	1.89 (0.96–3.73)	0.066
Tertiary	0.62 (0.16–2.37)	0.488
Cell count (cells/mm^3^)		
> 200	1.0 (ref)	
< 200	0.72 (0.31–1.67)	0.443
HIV duration (years)		
< 5	1.0 (ref)	
5–9	1.28 (0.64–2.57)	0.488
10 years and above	1.41 (0.54–3.66)	0.477

*significant *p* value of less than 0.05

## Discussion

The purpose of this study was to determine the prevalence of undetected psychiatric disorders among a low-income urban informal settlement population receiving comprehensive care for HIV/AIDS at a government-sponsored Health Centre in Nairobi. We found that 71% of patients, who were not currently receiving psychiatric treatment, had a psychiatric disorder needing an integrated management. The principal correlate of psychiatric disorders was low income, being of older age, and being a female.

Our findings resonate with other HIV studies reported in SSA. The reported prevalence of common mental disorders among HIV-infected people in low-income countries is variable. Literature points to ranges from 82.6% in Mulago Hospital in Kampala, Uganda [18], 63% in Yaounde, Cameroon, [19] 38.3% in Nigeria [20] to 11.2% in Dilla Hospital in south region of Ethiopia [21].

Depression is the most common mental health co-morbidity experienced by people living with HIV; the prevalence of depression among people with HIV is around 8–57% [22]. Our study found a prevalence of 32.2% of depression in our sample. The high prevalence found in studies such as North Central Nigeria in 2013 with 56.7% [23] and a study from three hospitals in Tigray region, Ethiopia, with 43.9% [24] may be due to different tools that were used and may also be attributed to childhood trauma, negative life events, and less social support factors that were not investigated in this study but may impact depression severity.

## Nature of previously undetected psychiatric morbidity

The leading co-morbidities were MDD (32.2%), PTSD (18.4%), Dysthymia (17.6%), and OCD (17.6%). This concurs with prevalence studies from LMIC which have reported rates of psychiatric disorder based on diagnostic interviews or psychiatric symptom scales.

Mental or psychological disorders often seem to be related to the severity of HIV/AIDS, medication side effects, younger age, higher viral load, the loss of health, a decrease in functioning, the deterioration of bodily integrity, anticipatory loss of life, pain, poor family support, and presence of AIDS in the spouse, and cultural factors like stigma, relative lack of appropriate treatment facilities, poor access to health care, and low educational level could have significantly contributed [25]. Though factors that could explain varying levels of exposure to negative life events, e.g., conflict, were not investigated in this study, the impact of negative life events in an HIV-positive individual’s life can be enormous and these risk factors whether distal or proximal need to be understood further.

Generalized anxiety disorder had a low prevalence of 2.5%. Our study finds a lower prevalence estimate as compared to a study done in Kenyatta National Hospital (KNH) CCC on HIV/AIDS adult patients [26] which found a prevalence of anxiety to be 77.25%. A possible explanation for the lower prevalence in this study could be that the study tools used were different. In the KNH study, Becks Anxiety Inventory Scale was used while this study used the MINI plus to assess anxiety. Another possible explanation is that new patients were excluded from the study and it is often found that anxiety is prominent following initial HIV diagnosis and anxiety symptoms can frequently recur and escalate in response to disease progression [27]. In our study, approximately 60% (*n* = 147) of patients with psychiatric illness had at least two existing psychiatric morbidities.

Additionally, the current study was carried out in a resource-constrained setting where it is conceivable that issues related to purchasing power associated with medication, poor nutrition, and poverty may have significantly contributed to the reduced psychosocial well-being of patients which in turn contributed to the presence of psychiatric morbidity.

It is in this regard that WHO 2001 [28], recommends that attention to the psychosocial needs of people with AIDS should be an integral part of HIV care. This includes assistance with employment, income, housing, informed decision making, coping with illness and discrimination, and prevention and treatment of mild and serious mental health problems [28].

There were several limitations to our study; first and foremost, the researcher was not able to collect comprehensive HIV morbidity-related data, explore social determinants in detail, and follow up on those diagnosed with psychiatric disorders. The cross-sectional design also presents a limitation with regards to determining causal associations. This work was also part of a postgraduate thesis project and therefore had narrow focus. Factors that could explain varying levels of exposure to negative life events, e.g., conflict, were not investigated in this study. The impact of negative life events in an HIV-positive individual’s life can be enormous and these risk factors whether distal or proximal need to be understood further. Follow-up was outside the domain of the current study. It is also likely that the mental illness in our participants was due to HIV or prior adverse experiences which we were not able to sufficiently study.

It was also difficult to tell whether the psychiatric morbidity was due to the side effects of ARVs that the patients were taking or HIV-related stigma or adversities that the patients may have been experiencing in their families, communities, or interaction with the health facility. This work was part of the first authors’ Master of Medicine degree in Psychiatry, therefore, due to the short duration of the study in which it had to be completed, it was not possible to follow up patients diagnosed with mental illness after initiation of treatment.

Despite these shortcomings we believe our study contributes to the existing literature on psychiatric morbidity in adult HIV population in Sub-Saharan Africa as few studies have specifically focused on undetected psychiatric morbidity. The study also used a validated and reliable screening tool as opposed to clinical diagnosis that is widespread, yielding robust estimates of the prevalence of psychiatric illness. The external validity of our study is high because it was conducted in the primary care setting where the majority of HIV/AIDS patients receive care.

## Conclusion

The results of this study show that patients with HIV/AIDS receiving follow-up care experience considerable undetected psychiatric morbidity. The most important associated features of major depression disorder were low socioeconomic status and female sex. Since the psychiatric morbidity had been previously undetected, there is need to provide holistic care including screening for mental well-being all through the treatment of HIV patients in low-income settings. The occurrence of psychiatric symptoms at different stages of disease is poorly defined and therefore focus should be put on the psychiatric manifestations in relation to the CD4 counts. More rigorous research is needed to put mental health services for people living with HIV/AIDS in Africa on the healthcare agenda.
